# Supplementary material for: Acceptance of evolution by high school students: Is religion the key factor?
Source: PLoS One. 2022 Sep 19;17(9):e0273929. doi: 10.1371/journal.pone.0273929 (PMC9484648; doi:10.1371/journal.pone.0273929)
Supplement: S1 Table — (DOCX) [file pone.0273929.s001.docx]

**S1 Table. General profile of items by questionnaire, with Chi-square tests comparing Brazilian and Italian students (all students comprised).**

|  | | | **Brazil** | **Italy** | **p-value**  **(Chi-Square)** |
| --- | --- | --- | --- | --- | --- |
| **G75** | True | N | 942 | 2477 | **< 0.0005** |
|  |  | Perc. (%) | 46.80% | 71.96% |  |
|  | False | N | 191 | 361 |  |
|  |  | Perc. (%) | 9.49% | 10.49% |  |
|  | Would not know | N | 880 | 604 |  |
|  |  | Perc. (%) | 43.72% | 17.55% |  |
|  | Total | N | 2013 | 3442 |  |
|  |  | Perc. (%) | 100.00% | 100.00% |  |
| **G76** | True | N | 1655 | 3299 | **< 0.0005** |
|  |  | Perc. (%) | 81.97% | 94.72% |  |
|  | False | N | 127 | 74 |  |
|  |  | Perc. (%) | 6.29% | 2.12% |  |
|  | Would not know | N | 237 | 110 |  |
|  |  | Perc. (%) | 11.74% | 3.16% |  |
|  | Total | N | 2019 | 3483 |  |
|  |  | Perc. (%) | 100.00% | 100.00% |  |
| **G77** | True | N | 1283 | 3001 | **< 0.0005** |
|  |  | Perc. (%) | 63.83% | 86.29% |  |
|  | False | N | 250 | 171 |  |
|  |  | Perc. (%) | 12.44% | 4.92% |  |
|  | Would not know | N | 477 | 306 |  |
|  |  | Perc. (%) | 23.73% | 8.80% |  |
|  | Total | N | 2010 | 3478 |  |
|  |  | Perc. (%) | 100.00% | 100.00% |  |
| **G78** | True | N | 1351 | 2484 | **< 0.0005** |
|  |  | Perc. (%) | 67.35% | 71.79% |  |
|  | False | N | 224 | 260 |  |
|  |  | Perc. (%) | 11.17% | 7.51% |  |
|  | Would not know | N | 431 | 716 |  |
|  |  | Perc. (%) | 21.49% | 20.69% |  |
|  | Total | N | 2006 | 3460 |  |
|  |  | Perc. (%) | 100.00% | 100.00% |  |
| **G79** | True | N | 840 | 2920 | **< 0.0005** |
|  |  | Perc. (%) | 41.92% | 84.37% |  |
|  | False | N | 549 | 206 |  |
|  |  | Perc. (%) | 27.40% | 5.95% |  |
|  | Would not know | N | 615 | 335 |  |
|  |  | Perc. (%) | 30.69% | 9.68% |  |
|  | Total | N | 2004 | 3461 |  |
|  |  | Perc. (%) | 100.00% | 100.00% |  |
| **G80** | True | N | 575 | 679 | **< 0.0005** |
|  |  | Perc. (%) | 28.75% | 19.72% |  |
|  | False | N | 298 | 1006 |  |
|  |  | Perc. (%) | 14.90% | 29.21% |  |
|  | Would not know | N | 1127 | 1759 |  |
|  |  | Perc. (%) | 56.35% | 51.07% |  |
|  | Total | N | 2000 | 3444 |  |
|  |  | Perc. (%) | 100.00% | 100.00% |  |
| **G81** | True | N | 615 | 2136 | **< 0.0005** |
|  |  | Perc. (%) | 30.86% | 61.65% |  |
|  | False | N | 365 | 379 |  |
|  |  | Perc. (%) | 18.31% | 10.94% |  |
|  | Would not know | N | 1013 | 950 |  |
|  |  | Perc. (%) | 50.83% | 27.42% |  |
|  | Total | N | 1993 | 3465 |  |
|  |  | Perc. (%) | 100.00% | 100.00% |  |
| **G83** | True | N | 432 | 431 | **< 0.0005** |
|  |  | Perc. (%) | 21.60% | 12.42% |  |
|  | False | N | 765 | 2140 |  |
|  |  | Perc. (%) | 38.25% | 61.69% |  |
|  | Would not know | N | 803 | 898 |  |
|  |  | Perc. (%) | 40.15% | 25.89% |  |
|  | Total | N | 2000 | 3469 |  |
|  |  | Perc. (%) | 100.00% | 100.00% |  |
