# Supplementary material for: Acceptance of evolution by high school students: Is religion the key factor?
Source: PLoS One. 2022 Sep 19;17(9):e0273929. doi: 10.1371/journal.pone.0273929 (PMC9484648; doi:10.1371/journal.pone.0273929)
Supplement: S2 Table — (DOCX) [file pone.0273929.s002.docx]

**S2 Table. Proportion Column Tests for each item (all religions comprised).**

|  | | **Brazil 2014** | **Italy 2014** |  |  |  |
| --- | --- | --- | --- | --- | --- | --- |
|  |  | (A) | (B) |  |  |  |
| **G75** | True |  | **A(< 0.0005)** |  |  |  |
|  | False |  |  |  |  |  |
|  | Would not know | **B(< 0.0005)** |  |  |  |  |
| **G76** | True |  | **A(< 0.0005)** |  |  |  |
|  | False | **B(< 0.0005)** |  |  |  |  |
|  | Would not know | **B(< 0.0005)** |  |  |  |  |
| **G77** | True |  | **A(< 0.0005)** |  |  |  |
|  | False | **B(< 0.0005)** |  |  |  |  |
|  | Would not know | **B(< 0.0005)** |  |  |  |  |
| **G78** | True |  | **A(0.001)** |  |  |  |
|  | False | **B(< 0.0005)** |  |  |  |  |
|  | Would not know |  |  |  |  |  |
| **G79** | True |  | **A(< 0.0005)** |  |  |  |
|  | False | **B(< 0.0005)** |  |  |  |  |
|  | Would not know | **B(< 0.0005)** |  |  |  |  |
| **G80** | True | **B(< 0.0005)** |  |  |  |  |
|  | False |  | **A(< 0.0005)** |  |  |  |
|  | Would not know | **B(< 0.0005)** |  |  |  |  |
| **G81** | True |  | **A(< 0.0005)** |  |  |  |
|  | False | **B(< 0.0005)** |  |  |  |  |
|  | Would not know | **B(< 0.0005)** |  |  |  |  |
| **G83** | True | **B(< 0.0005)** |  |  |  |  |
|  | False |  | **A(< 0.0005)** |  |  |  |
|  | Would not know | **B(< 0.0005)** |  |  |  |  |
