# Supplementary material for: Acceptance of evolution by high school students: Is religion the key factor?
Source: PLoS One. 2022 Sep 19;17(9):e0273929. doi: 10.1371/journal.pone.0273929 (PMC9484648; doi:10.1371/journal.pone.0273929)
Supplement: S3 Table — MCA case processing summary. (DOCX) [file pone.0273929.s003.docx]

**S3 Table. Summary of the Multiple Correspondence Analysis.** MCA Case Processing Summary.

The Case Processing Summary table shows that only 772 records had valid responses for all variables in the analysis, and 3,109 records had a missing value for at least one variable. All of these records, except the 63 cases with missing values in all items, were included in the analysis, totaling 3,881 cases. Processing stopped at the ninth iteration soon after reaching the convergence test value.

| Valid active cases | 772 |
| --- | --- |
| Active cases with missing values | 3,109 |
| Complimentary cases | 63 |
| Total | 3,944 |
| Cases included in the analysis | 3,881 |
