# Supplementary material for: Acceptance of evolution by high school students: Is religion the key factor?
Source: PLoS One. 2022 Sep 19;17(9):e0273929. doi: 10.1371/journal.pone.0273929 (PMC9484648; doi:10.1371/journal.pone.0273929)
Supplement: S4 Table — (DOCX) [file pone.0273929.s004.docx]

**S4 Table. Sample of students who declared their religion in Italy and Brazil.**

The sample of students who declared their religion can be seen in Table S4. As the number of Italian non-Catholic Christians was low, they were not included in closer comparisons regarding the Intercultural Index.

| Brazilian Catholics | 986 |
| --- | --- |
| Italian Catholics | 2,296 |
| Brazilian Non-Catholic Christians | 560 |
| Italian Non-Catholic Christians | 102 |
| Total | 3,944 |
