# Supplementary material for: Acceptance of evolution by high school students: Is religion the key factor?
Source: PLoS One. 2022 Sep 19;17(9):e0273929. doi: 10.1371/journal.pone.0273929 (PMC9484648; doi:10.1371/journal.pone.0273929)
Supplement: S5 Table — (DOCX) [file pone.0273929.s005.docx]

**S5 Table. MCA Matrix of Discrimination Measures.**

The matrix of discrimination measures (Table S5) quantifies the degree of association of each item with the dimensions of the analysis, similar to an R-square measure of linear regression. A high number shows a high association between the item and the respective dimension. These measures assume positive values and can be greater than 1 when there are missing values in the database, which is the case of both databases in Brazil and Italy. The lowest association with both dimensions was seen with item G80 (see main text).

| **ITEM** | **DIMENSION 1** | **DIMENSION 2** | **MEAN** |
| --- | --- | --- | --- |
| G75 | 0.877 | 0.942 | 0.909 |
| G76 | 1.368 | 1.334 | 1.351 |
| G77 | 1.208 | 1.191 | 1.199 |
| G79 | 1.126 | 1.101 | 1.114 |
| G80 | 0.701 | 0.644 | 0.673 |
| G81 | 0.770 | 0.846 | 0.808 |
| G83 | 0.950 | 0.941 | 0.945 |
| **Total Assets** | **7.000** | **7.000** | **7.000** |

The results show that the two dimensions have roughly the same weight within the analysis. All items have similar measures in the two dimensions, indicating that they have similar degrees of association with each item.
