# Supplementary material for: Acceptance of evolution by high school students: Is religion the key factor?
Source: PLoS One. 2022 Sep 19;17(9):e0273929. doi: 10.1371/journal.pone.0273929 (PMC9484648; doi:10.1371/journal.pone.0273929)
Supplement: S1 File — (DOCX) [file pone.0273929.s006.docx]

**S1 File. Reasons to consider item G78 as an outlier.**

Brazilian and Italian Catholics showed significant differences in all items except G78 ("Evolution occurs in both plants and animals"), which was a rephrased version of the previous item (G77, "Present-day species of animals and plants originated from other species of the past"). All students answered the items in the same numeric order. Brazilian Catholics' answers were very similar in the two items, but there was an important change in the Italian side, as 1946 (85.24%) students took G77 as true but this number dropped to 1597 (70.45%) in the following item.

The inclusion of the word “evolution” in G78 may have added some noise in the testing, possibly inducing Italian Catholics to take it as an examination trap, which students use to be trained to figure out in multiple-choice tests. The number of Italians who preferred not to answer raised from 219 (9,59%) in G77 to 497 (21,92%) in G78.

This change led to a lack of significance in the difference of the positive answers of G78. Thus, these results were taken as outliers for statistical purposes, and the item was not considered for both MCA (Fig 1, main text) as well as for the Intercultural Index (Table 5 and Fig 2, main text). However, G78 also had an Intercultural Index higher than 1 (1,16).

The broad picture (Tables S1 and S2) shows very significant differences between Italy and Brazil (p-value <0.0005). In general, Italians tend to believe more in the statements about evolution than Brazilians, except for item G80 (“Human species has inhabited the Earth for about 100,000 years”). The reasons to consider G80 results in both analyses are discussed in the main text, and Table S5 shows that the behavior of the answers was different, with the lowest level of association with both dimensions.
