# Supplementary material for: Acceptance of evolution by high school students: Is religion the key factor?
Source: PLoS One. 2022 Sep 19;17(9):e0273929. doi: 10.1371/journal.pone.0273929 (PMC9484648; doi:10.1371/journal.pone.0273929)
Supplement: S2 File — (DOCX) [file pone.0273929.s007.docx]

S2 File – Study of missing cases in the Multiple Correspondence Analysis

The missing values analysis was performed by comparing the three groups of Multiple Correspondence Analysis (MCA) cases (valid active cases, cases with missing values and complementary cases) with the country in which the questionnaire was applied (Brazil/Italy), the religious group (Catholic/Christian non-Catholic) declared by the respondents and the combination of the two factors (country of application versus religious group). In the three cases (Tables S6.1 to S6.3), there is no significant bias between the distributions of the two principal MCA groups. It should be taken into account that the complementary cases comprise those with missing values for all items and were not computed for the MCA output (only 63 cases).

|  | **Table S6.1: Distribution of cases by country** | | |  |
| --- | --- | --- | --- | --- |
|  | Case Identification | | |  |
|  | Active valid cases | Active cases with missing values | Complementary cases | TOTAL |
| N | 178 | 1325 | 43 | 1,546 |
| Brazil  Pct. (%) | 23.06% | 42.62% | 68.25% | 39.20% |
| N | 594 | 1,784 | 20 | 2,398 |
| Italy  Pct. (%) | 76.94% | 57.38% | 31.75% | 60.80% |
| N | 772 | 3,109 | 63 | 3,944 |
| TOTAL  Pct. (%) | 100.00% | 100.00% | 100.00% | 100.00% |

|  | **Table S6.2: Distribution of cases by religious group** | | | | |  |
| --- | --- | --- | --- | --- | --- | --- |
|  |  | | Case Identification | | |  |
|  |  |  | Active valid cases | Active cases with missing values | Complementary cases | TOTAL |
| Religious Group | Catholic | N | 685 | 2,550 | 47 | 3,282 |
|  |  | Pct. (%) | 88.73% | 82.02% | 74.60% | 83.22% |
|  | Non-Catholic  Christian | N | 87 | 559 | 16 | 662 |
|  |  | Pct. (%) | 11.27% | 17.98% | 25.40% | 16.78% |
|  | TOTAL | N | 772 | 3,109 | 63 | 3,944 |
|  |  | Pct. (%) | 100.00% | 100.00% | 100.00% | 100.00% |

|  | **Table S6.3: Distribution of cases by country and religious group** | | | | |  |
| --- | --- | --- | --- | --- | --- | --- |
|  |  | | Case Identification | | |  |
|  |  |  | Active valid cases | Active cases with missing values | Complementary cases | TOTAL |
| Brazil | Catholic | N | 114 | 843 | 29 | 986 |
|  |  | Pct. (%) | 64.04% | 63.62% | 67.44% | 63.78% |
|  | Non-  Catholic  Christian | N | 64 | 482 | 14 | 560 |
|  |  | Pct. (%) | 35.96% | 36.38% | 32.56% | 36.22% |
|  | TOTAL | N | 178 | 1,325 | 43 | 1,546 |
|  |  | Pct. (%) | 100.00% | 100.00% | 100.00% | 100.00% |
| Italy) | Catholic | N | 571 | 1,707 | 18 | 2,296 |
|  |  | Pct. (%) | 96.13% | 95.68% | 90.00% | 95.75% |
|  | Non-  Catholic  Christian | N | 23 | 77 | 2 | 102 |
|  |  | Pct. (%) | 3.87% | 4.32% | 10.00% | 4.25% |
|  | TOTAL | N | 594 | 1,784 | 20 | 2,398 |
|  |  | Pct. (%) | 100.00% | 100.00% | 100.00% | 100.00% |
| TOTAL | Catholic | N | 685 | 2,550 | 47 | 3,282 |
|  |  | Pct. (%) | 88.73% | 82.02% | 74.60% | 83.22% |
|  | Non-  Catholic  Christian | N | 87 | 559 | 16 | 662 |
|  |  | Pct. (%) | 11.27% | 17.98% | 25.40% | 16.78% |
|  | TOTAL | N | 772 | 3,109 | 63 | 3,944 |
|  |  | Pct. (%) | 100.00% | 100.00% | 100.00% | 100.00% |

Each of the items used for the MCA study was analyzed (Tables S6.4 to S6.10), comparing only valid active cases and cases with missing values. No group of valid cases (eg, Brazilian Catholics who responded true - BC+) was dominant, while another group was dominant in the opposite direction, with absent values (eg, Italian Catholics who responded false - IC-).

| **Table S6.4: Distribution of G75 by country, religious group and case identification** | | | | |
| --- | --- | --- | --- | --- |
|  | | Case Identification | | |
|  |  | Active valid cases | Active cases with missing values | TOTAL |
| G75 | BC+ | 100 | 365 | 465 |
|  |  | 12.95% | 17.96% | 16.58% |
|  | BC- | 14 | 63 | 77 |
|  |  | 1.81% | 3.10% | 2.75% |
|  | BNC+ | 46 | 179 | 225 |
|  |  | 5.96% | 8.81% | 8.02% |
|  | BNC- | 18 | 51 | 69 |
|  |  | 2.33% | 2.51% | 2.46% |
|  | IC+ | 482 | 1,177 | 1,659 |
|  |  | 62.44% | 57.92% | 59.17% |
|  | IC- | 89 | 143 | 232 |
|  |  | 11.53% | 7.04% | 8.27% |
|  | INC+ | 20 | 45 | 65 |
|  |  | 2.59% | 2.21% | 2.32% |
|  | INC- | 3 | 9 | 12 |
|  |  | 0.39% | 0.44% | 0.43% |
|  | TOTAL | 772 | 2,032 | 2,804 |
|  |  | 100.00% | 100.00% | 100.00% |

| **Table S6.5: Distribution of G76 by country, religious group and case identification** | | | | |
| --- | --- | --- | --- | --- |
|  | | Case Identification | | |
|  |  | Active valid cases | Active cases with missing values | TOTAL |
| G76 | BC+ | 107 | 714 | 821 |
|  |  | 13.86% | 24.68% | 22.40% |
|  | BC- | 7 | 43 | 50 |
|  |  | 0.91% | 1.49% | 1.36% |
|  | BNC+ | 58 | 374 | 432 |
|  |  | 7.51% | 12.93% | 11.79% |
|  | BNC- | 6 | 36 | 42 |
|  |  | 0.78% | 1.24% | 1.15% |
|  | IC+ | 559 | 1,616 | 2,175 |
|  |  | 72.41% | 55.86% | 59.35% |
|  | IC- | 12 | 37 | 49 |
|  |  | 1.55% | 1.28% | 1.34% |
|  | INC+ | 22 | 71 | 93 |
|  |  | 2.85% | 2.45% | 2.54% |
|  | INC- | 1 | 2 | 3 |
|  |  | 0.13% | 0.07% | 0.08% |
|  | TOTAL | 772 | 2,893 | 3,665 |
|  |  | 100.00% | 100.00% | 100.00% |

| **Table S6.6: Distribution of G77 by country, religious group and case identification** | | | | |
| --- | --- | --- | --- | --- |
|  | | Case Identification | | |
|  |  | Active valid cases | Active cases with missing values | TOTAL |
| G77 | BC+ | 94 | 547 | 641 |
|  |  | 12.18% | 21.56% | 19.37% |
|  | BC- | 20 | 75 | 95 |
|  |  | 2.59% | 2.96% | 2.87% |
|  | BNC+ | 42 | 282 | 324 |
|  |  | 5.44% | 11.12% | 9.79% |
|  | BNC- | 22 | 71 | 93 |
|  |  | 2.85% | 2.80% | 2.81% |
|  | IC+ | 537 | 1,409 | 1,946 |
|  |  | 69.56% | 55.54% | 58.81% |
|  | IC- | 34 | 84 | 118 |
|  |  | 4.40% | 3.31% | 3.57% |
|  | INC+ | 21 | 59 | 80 |
|  |  | 2.72% | 2.33% | 2.42% |
|  | INC- | 2 | 10 | 12 |
|  |  | 0.26% | 0.39% | 0.36% |
|  | TOTAL | 772 | 2,537 | 3,309 |
|  |  | 100.00% | 100.00% | 100.00% |

| **Table S6.7: Distribution of G79 by country, religious group and case identification** | | | | |
| --- | --- | --- | --- | --- |
|  | | Case Identification | | |
|  |  | Active valid cases | Active cases with missing values | TOTAL |
| G79 | BC+ | 80 | 386 | 466 |
|  |  | 10.36% | 15.88% | 14.55% |
|  | BC- | 34 | 172 | 206 |
|  |  | 4.40% | 7.08% | 6.43% |
|  | BNC+ | 23 | 138 | 161 |
|  |  | 2.98% | 5.68% | 5.03% |
|  | BNC- | 41 | 184 | 225 |
|  |  | 5.31% | 7.57% | 7.03% |
|  | IC+ | 529 | 1,397 | 1,926 |
|  |  | 68.52% | 57.49% | 60.15% |
|  | IC- | 42 | 91 | 133 |
|  |  | 5.44% | 3.74% | 4.15% |
|  | INC+ | 21 | 45 | 66 |
|  |  | 2.72% | 1.85% | 2.06% |
|  | INC- | 2 | 17 | 19 |
|  |  | 0.26% | 0.70% | 0.59% |
|  | TOTAL | 772 | 2,430 | 3,202 |
|  |  | 100.00% | 100.00% | 100.00% |

| **Table S6.8: Distribution of G80 by country, religious group and case identification** | | | | |
| --- | --- | --- | --- | --- |
|  | | Case Identification | | |
|  |  | Active valid cases | Active cases with missing values | TOTAL |
| G80 | BC+ | 67 | 198 | 265 |
|  |  | 8.68% | 18.82% | 14.53% |
|  | BC- | 47 | 102 | 149 |
|  |  | 6.09% | 9.70% | 8.17% |
|  | BNC+ | 38 | 122 | 160 |
|  |  | 4.92% | 11.60% | 8.77% |
|  | BNC- | 26 | 58 | 84 |
|  |  | 3.37% | 5.51% | 4.61% |
|  | IC+ | 196 | 255 | 451 |
|  |  | 25.39% | 24.24% | 24.73% |
|  | IC- | 375 | 293 | 668 |
|  |  | 48.58% | 27.85% | 36.62% |
|  | INC+ | 6 | 10 | 16 |
|  |  | 0.78% | 0.95% | 0.88% |
|  | INC- | 17 | 14 | 31 |
|  |  | 2.20% | 1.33% | 1.70% |
|  | TOTAL | 772 | 1,052 | 1,824 |
|  |  | 100.00% | 100.00% | 100.00% |

| **Table S6.9: Distribution of G81 by country, religious group and case identification** | | | | |
| --- | --- | --- | --- | --- |
|  | | Case Identification | | |
|  |  | Active valid cases | Active cases with missing values | TOTAL |
| G81 | BC+ | 72 | 224 | 296 |
|  |  | 9.33% | 13.53% | 12.20% |
|  | BC- | 42 | 126 | 168 |
|  |  | 5.44% | 7.61% | 6.92% |
|  | BNC+ | 35 | 116 | 151 |
|  |  | 4.53% | 7.01% | 6.22% |
|  | BNC- | 29 | 79 | 108 |
|  |  | 3.76% | 4.77% | 4.45% |
|  | IC+ | 483 | 887 | 1,370 |
|  |  | 62.56% | 53.60% | 56.45% |
|  | IC- | 88 | 172 | 260 |
|  |  | 11.40% | 10.39% | 10.71% |
|  | INC+ | 16 | 39 | 55 |
|  |  | 2.07% | 2.36% | 2.27% |
|  | INC- | 7 | 12 | 19 |
|  |  | 0.91% | 0.73% | 0.78% |
|  | TOTAL | 772 | 1,655 | 2,427 |
|  |  | 100.00% | 100.00% | 100.00% |

| **Table S6.10: Distribution of G83 by country, religious group and case identification** | | | | |
| --- | --- | --- | --- | --- |
|  | | Case Identification | | |
|  |  | Active valid cases | Active cases with missing values | TOTAL |
| G83 | BC+ | 42 | 178 | 220 |
|  |  | 5.44% | 9.53% | 8.34% |
|  | BC- | 72 | 274 | 346 |
|  |  | 9.33% | 14.68% | 13.11% |
|  | BNC+ | 20 | 85 | 105 |
|  |  | 2.59% | 4.55% | 3.98% |
|  | BNC- | 44 | 178 | 222 |
|  |  | 5.70% | 9.53% | 8.41% |
|  | IC+ | 79 | 214 | 293 |
|  |  | 10.23% | 11.46% | 11.10% |
|  | IC- | 492 | 886 | 1,378 |
|  |  | 63.73% | 47.46% | 52.22% |
|  | INC+ | 5 | 11 | 16 |
|  |  | 0.65% | 0.59% | 0.61% |
|  | INC- | 18 | 41 | 59 |
|  |  | 2.33% | 2.20% | 2.24% |
|  | TOTAL | 772 | 1,867 | 2,639 |
|  |  | 100.00% | 100.00% | 100.00% |

CONCLUSION:

The detailed analysis of the occurrence of missing values allows us to conclude that there is no evidence that the MCA results have any significant bias when comparing the responses of valid active cases with those with missing values.

The databank and the SPSS sintaxes for the MCA study and all other tables are available at: <https://github.com/easouza85/Nelio-Bizzo-Project>
